# Supplementary figures and images for: Target-specific requirements for RNA interference can arise through restricted RNA amplification despite the lack of specialized pathways
Source: eLife. 2024 Aug 20;13:RP97487. doi: 10.7554/eLife.97487 (PMC11335349; doi:10.7554/eLife.97487)

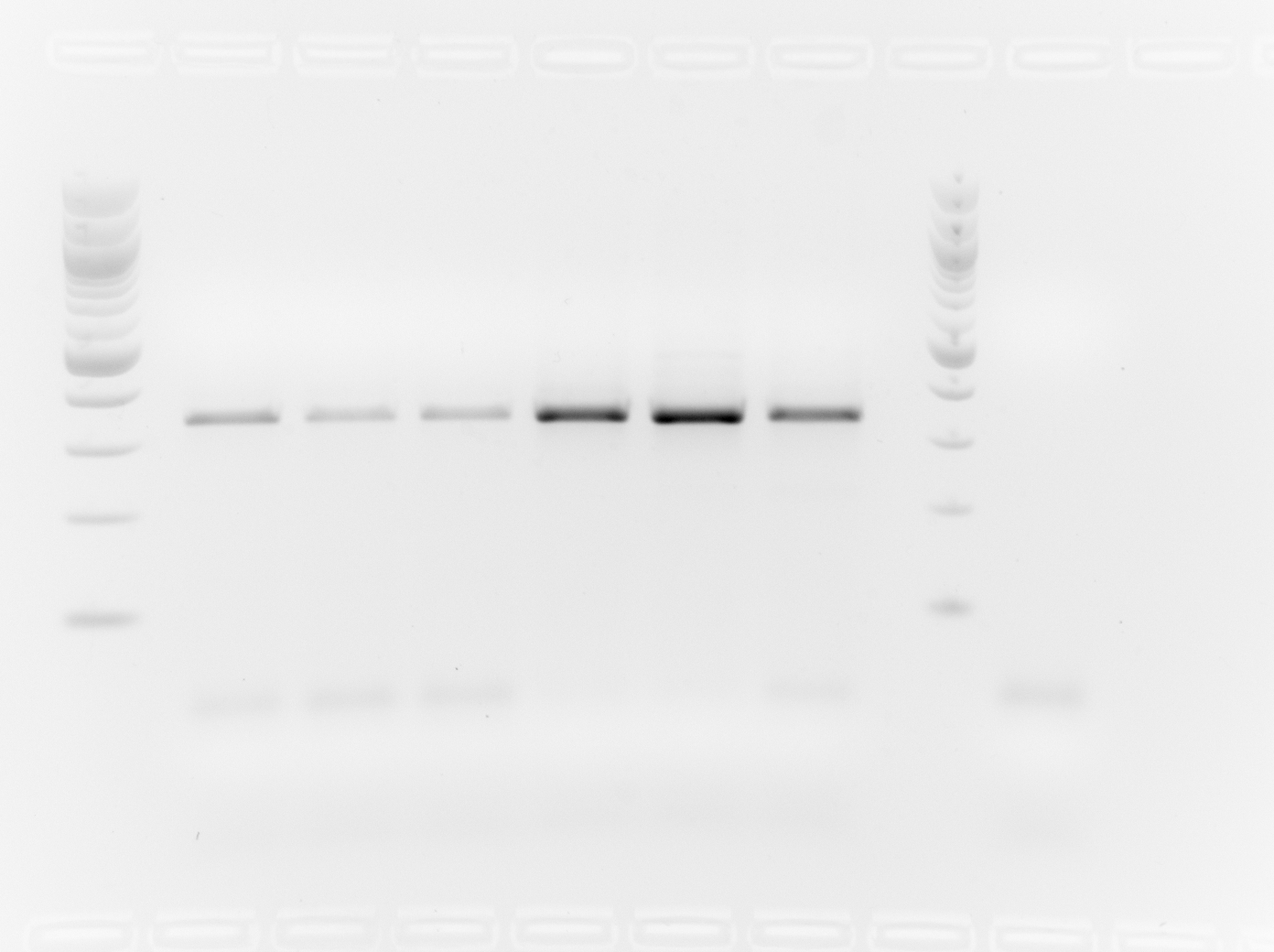

Supplement: Figure 3—figure supplement 1—source data 1. [file elife-97487-fig3-figsupp1-data1.tif]

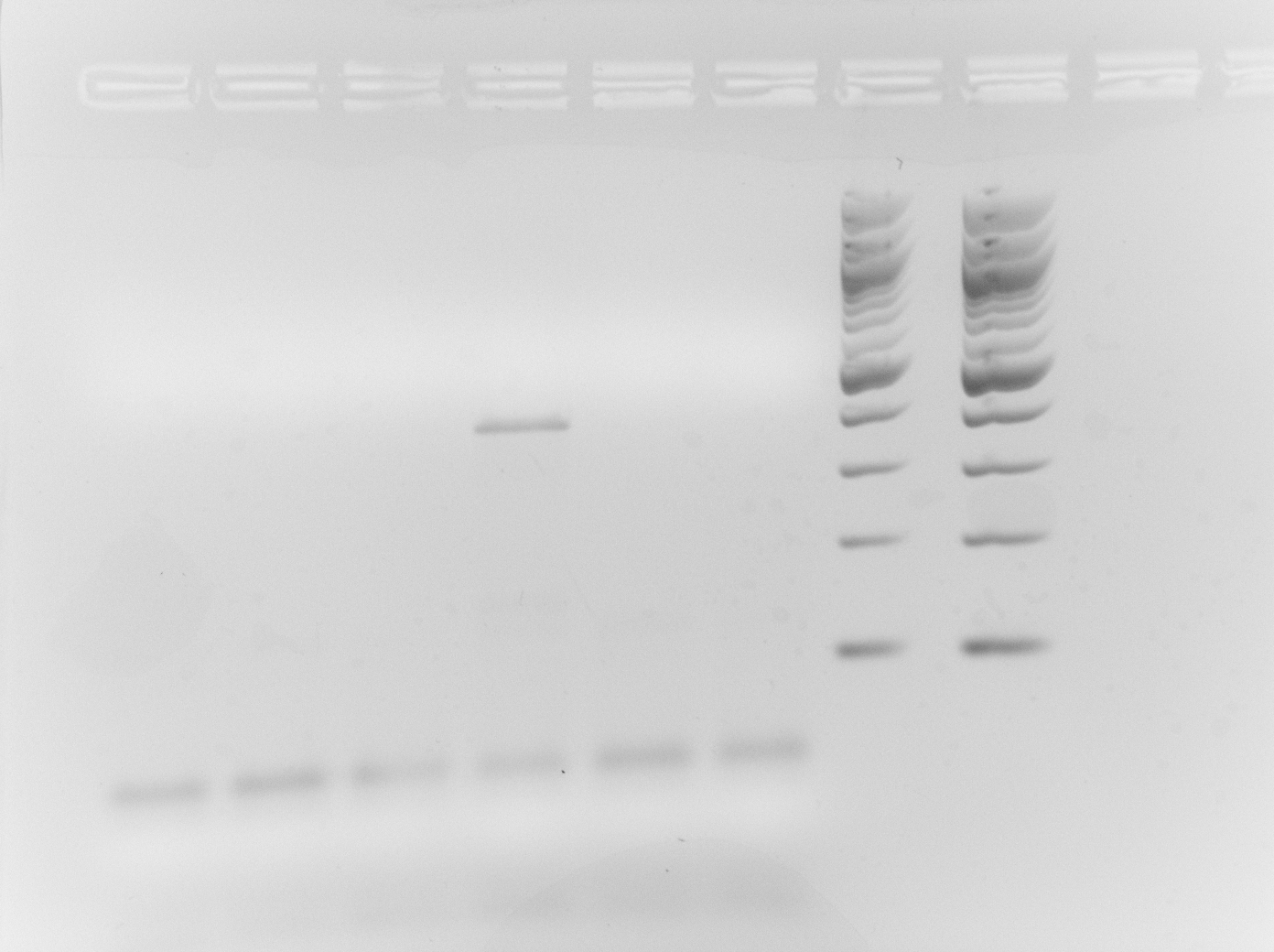

Supplement: Figure 3—figure supplement 1—source data 2. [file elife-97487-fig3-figsupp1-data2.tif]

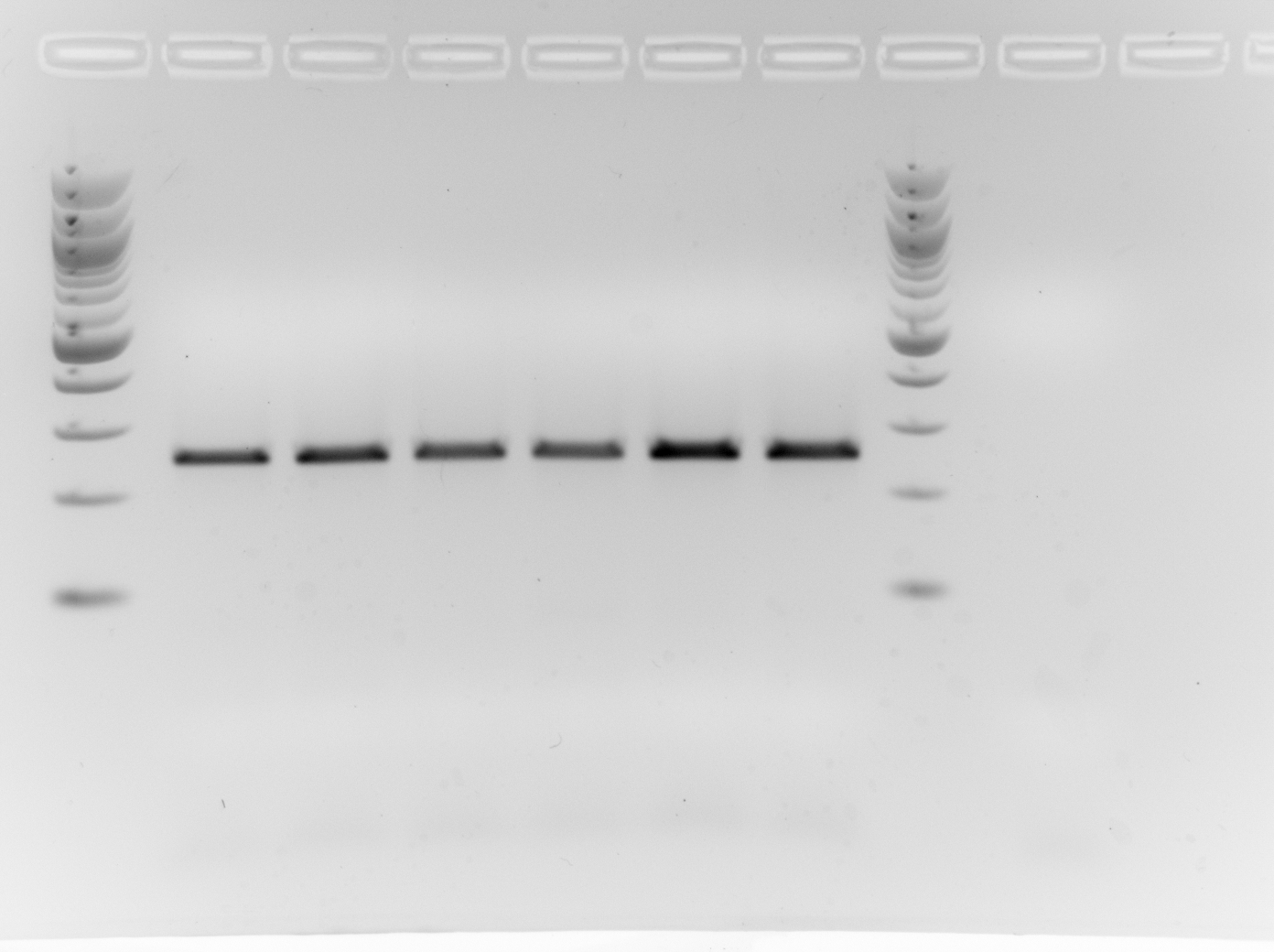

Supplement: Figure 3—figure supplement 1—source data 3. [file elife-97487-fig3-figsupp1-data3.tif]

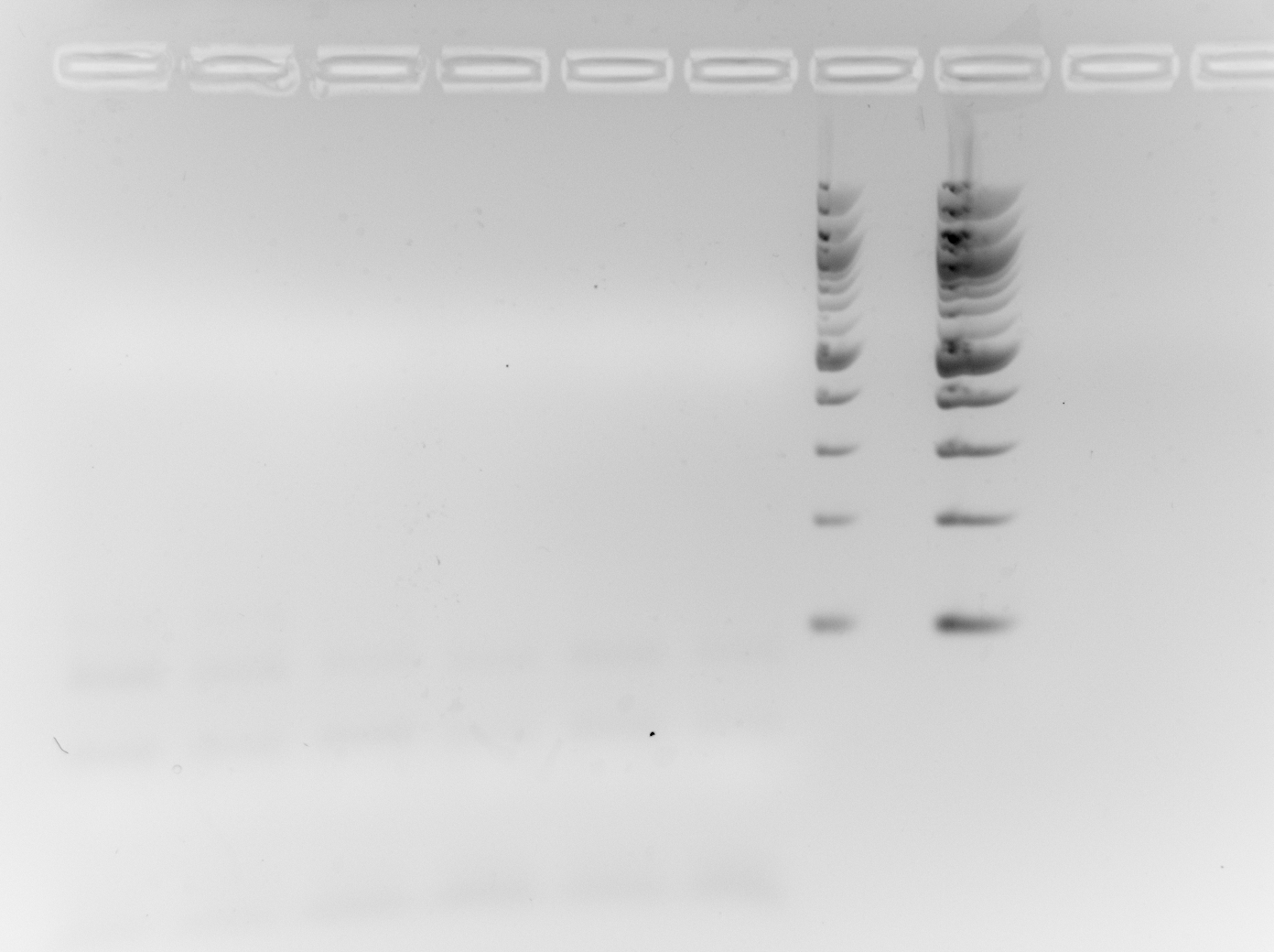

Supplement: Figure 3—figure supplement 1—source data 4. [file elife-97487-fig3-figsupp1-data4.tif]

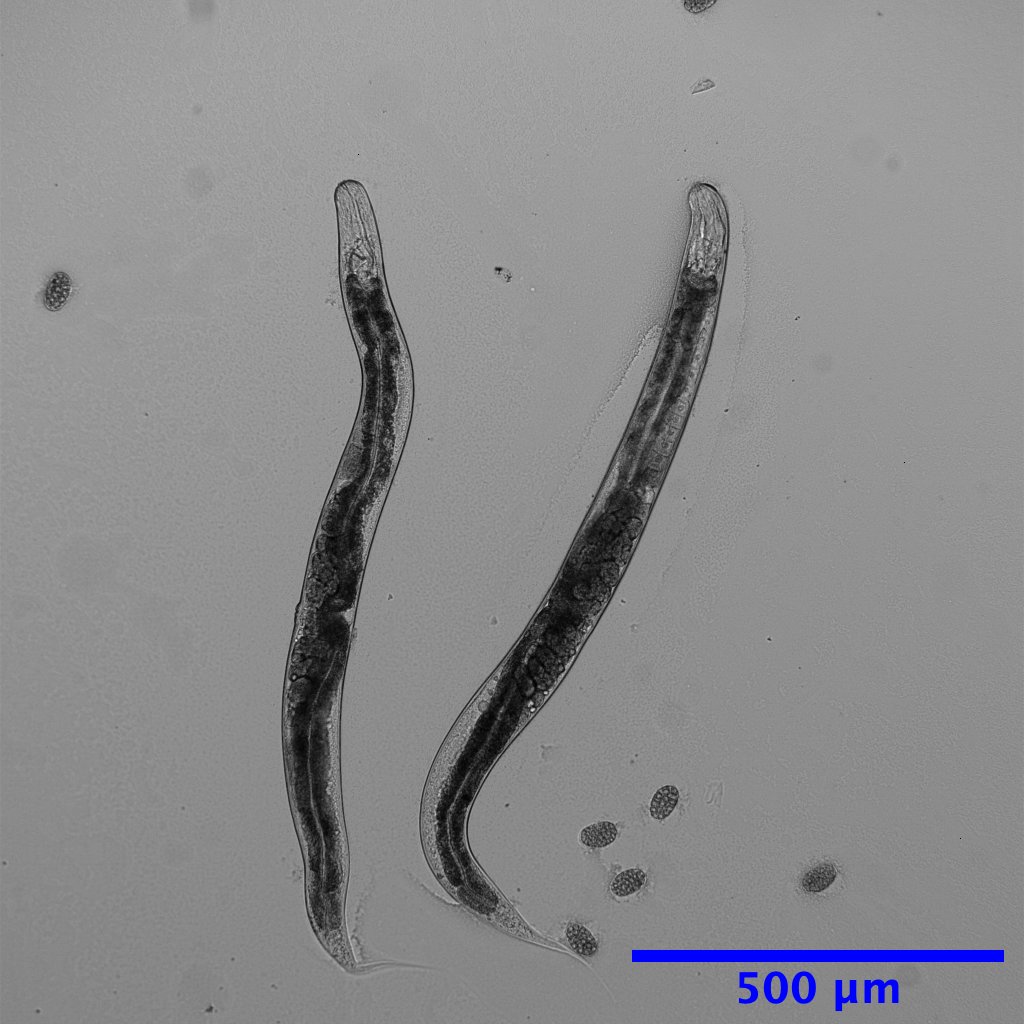

Supplement: Figure 5—source data 2. [file elife-97487-fig5-data2.zip › Fig5C_gfp_dic.jpg]

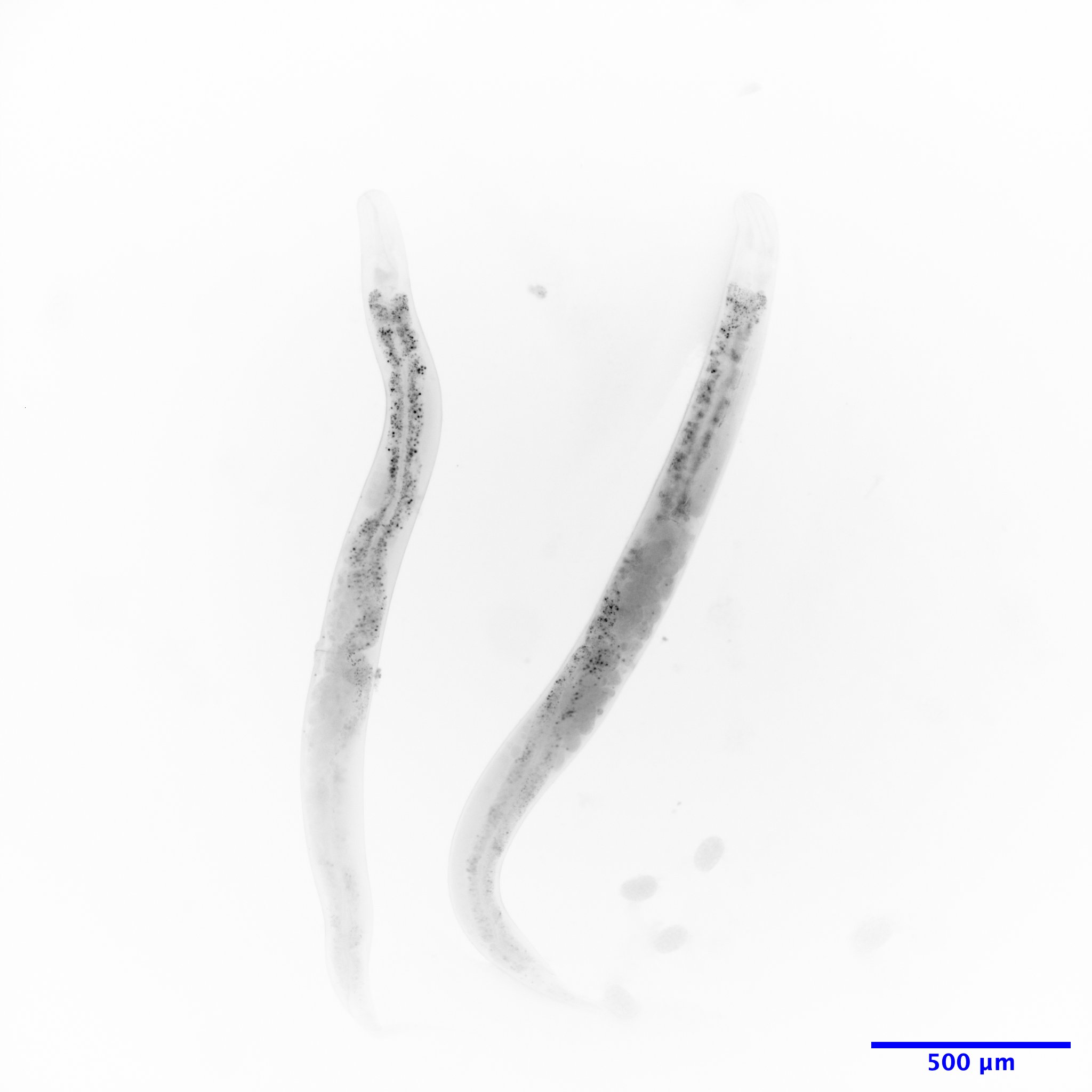

Supplement: Figure 5—source data 2. [file elife-97487-fig5-data2.zip › Fig5C_gfp_gfp.jpg]

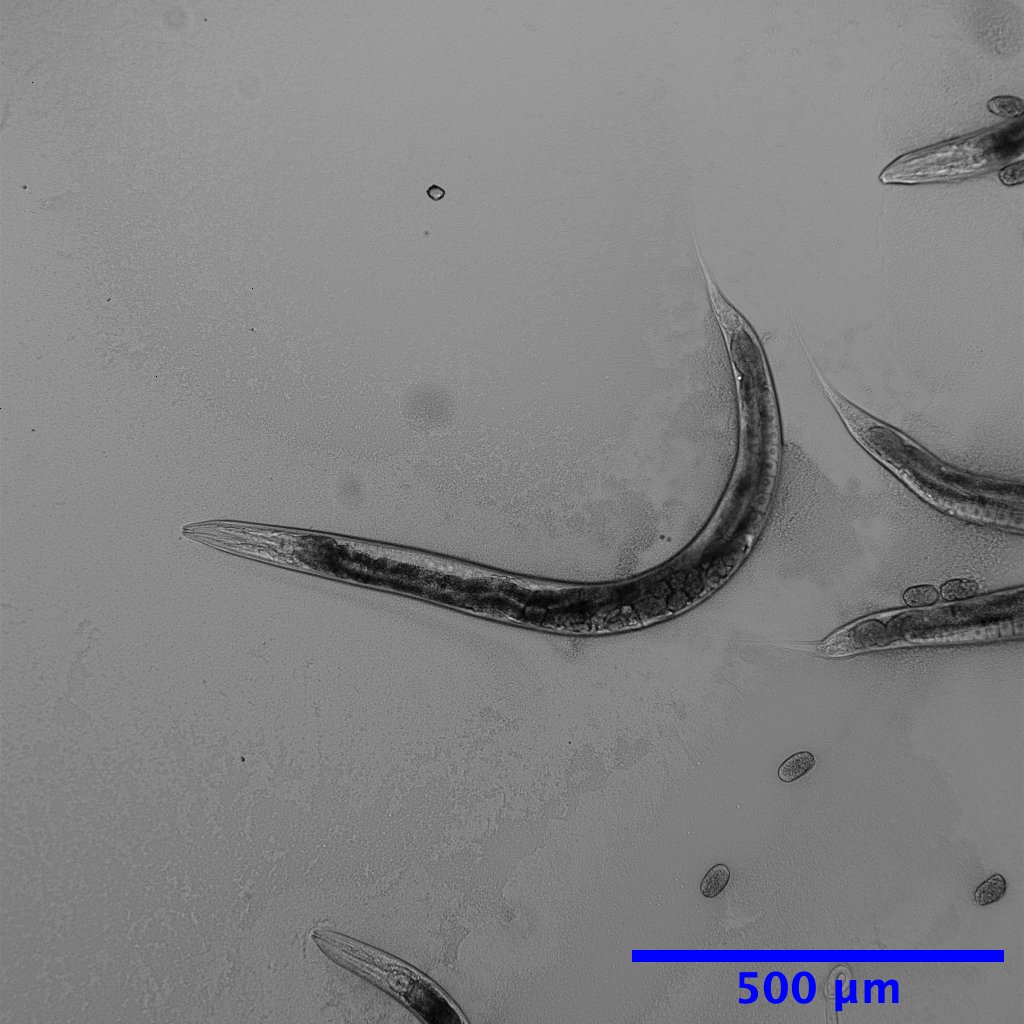

Supplement: Figure 5—source data 2. [file elife-97487-fig5-data2.zip › Fig5C_l4440.jpg]

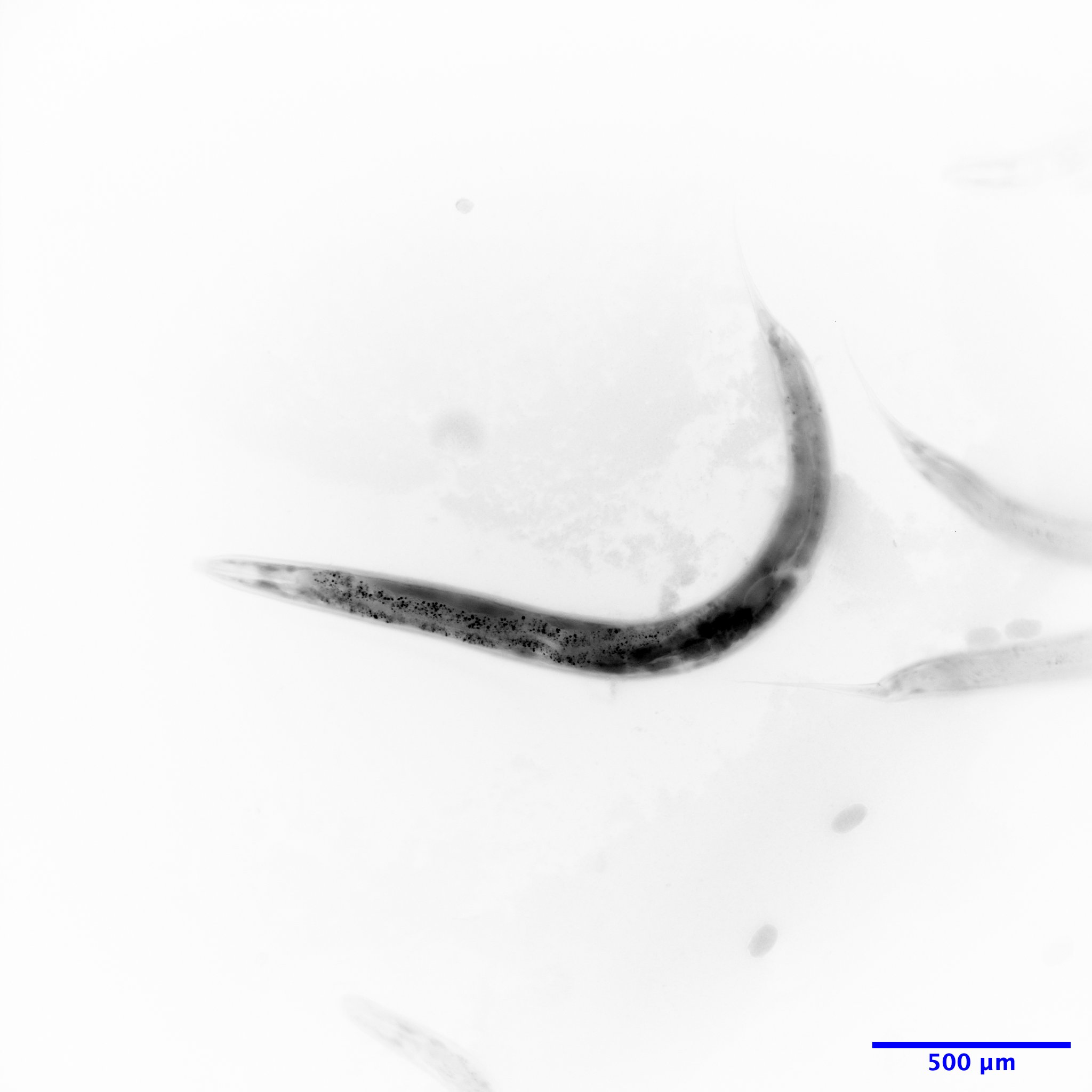

Supplement: Figure 5—source data 2. [file elife-97487-fig5-data2.zip › Figure 5C.jpg]

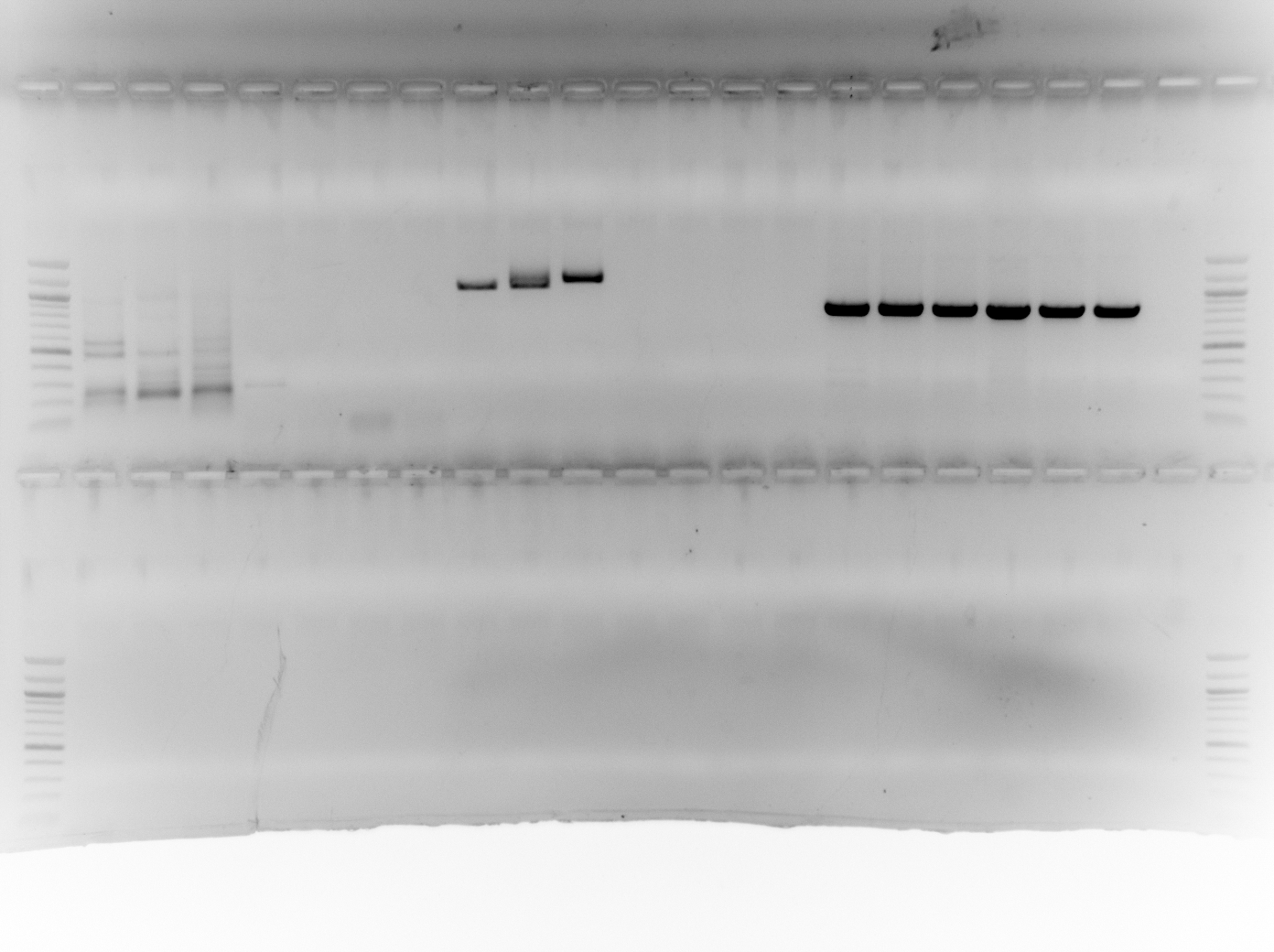

Supplement: Figure 6—source data 2. [file elife-97487-fig6-data2.png]
